# The *GNAS* Locus: Quintessential Complex Gene Encoding Gsα, XLαs, and other Imprinted Transcripts

**DOI:** 10.2174/138920207783406488

**Published:** 2007-09

**Authors:** Murat Bastepe

**Affiliations:** Endocrine Unit, Department of Medicine, Massachusetts General Hospital and Harvard Medical School, Boston, MA, USA

## Abstract

The currently estimated number of genes in the human genome is much smaller than previously predicted. As an explanation for this disparity, most individual genes have multiple transcriptional units that represent a variety of biologically important gene products. *GNAS* exemplifies a gene of such complexity. One of its products is the α-subunit of the stimulatory heterotrimeric G protein (Gsα), a ubiquitous signaling protein essential for numerous different cellular responses. Loss-of-function and gain-of-function mutations within Gsα-coding *GNAS* exons are found in various human disorders, including Albright’s hereditary osteodystrophy, pseudohypoparathyroidism, fibrous dysplasia of bone, and some tumors of different origin. While Gsα expression in most tissues is biallelic, paternal Gsα expression is silenced in a small number of tissues, playing an important role in the development of phenotypes associated with *GNAS* mutations. Additional products derived exclusively from the paternal *GNAS* allele include XLαs, a protein partially identical to Gsα, and two non-coding RNA molecules, the A/B transcript and the antisense transcript. The maternal *GNAS* allele leads to NESP55, a chromogranin-like neuroendocrine secretory protein. *In vivo* animal models have demonstrated the importance of each of the exclusively imprinted *GNAS* products in normal mammalian physiology. However, although one or more of these products are also disrupted by most naturally occurring *GNAS* mutations, their roles in disease pathogenesis remain unknown. To further our understanding of the significance of this gene in physiology and pathophysiology, it will be important to elucidate the cellular roles and the mechanisms regulating the expression of each *GNAS* product.

## INTRODUCTION

The first draft of the human genome DNA sequence was reported in 2001 independently by the Human Genome Project and Celera Genomics [1, 2]. Since this important landmark in medical research, the entire research community has had access to a wealth of information that would otherwise have taken many years to gather, resulting in a tremendous acceleration of virtually all research studies that involve human biology or disease. With updates of the project, the sequence is nearly complete [3, 4]. It is interesting, however, that the number of genes in the human genome appears to be much smaller than initially estimated. Although the exact number of genes in the genome still remains unknown, more recent estimate for the number of protein-coding genes is between 20,000 and 25,000 [5], which markedly differs from the previously predicted number of approximately 100,000 [6-9]. The currently estimated gene number, of course, appears quite small, considering the complexity of our species. However, the difference between the older and most recent predictions of the gene number can be accounted for by the presence of multiple transcriptional units associated with many individual genes. In fact, the total number of proteins encoded by the human genome may be similar to, if not higher than, the past estimates of the gene number. This review focuses on the *GNAS* locus, which exemplifies a complex gene with multiple gene products derived through alternative promoter use and alternative pre-mRNA splicing.

## THE COMPLEX *GNAS* LOCUS AND Gsα

**Fig. (1):**
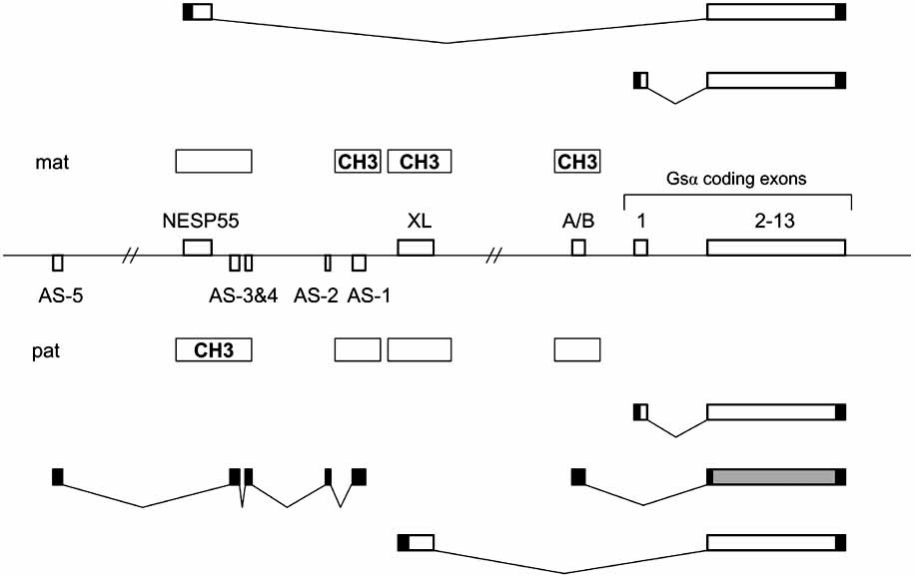
Multiple imprinted sense and antisense transcripts from the complex *GNAS* locus. Exons 1-13 encode Gsα , which is biallelic in most tissues; however, paternal Gsα  allele is silenced in a small number of tissues, including the renal proximal tubule, thyroid, and pituitary. From differentially methylated promoters arise several other transcripts, including the maternally expressed NESP55 and the paternally expressed XLαs. Both of these transcripts use individual first exons that splice onto exons 2-13. In addition, the paternal *GNAS* allele gives rise to a transcript termed A/B (also referred to as 1A or 1’), which also shares exons 2-13 and is presumed to be non-coding. Note that the A/B transcript contains an ORF (colored grey) that could lead to a translational product, but the existence of endogenous A/B protein is not supported experimentally. Another non-coding transcript is also derived from the paternal *GNAS* allele, but this transcript is made from the antisense strand (AS transcript). Boxes and connecting lines depict exons and introns, respectively. Open rectangles and rectangles filled with CH3 show non-methylated and methylated DMRs, respectively. The distance between AS exon 5 and exon NESP55 is ~19 kb and the distance between exon A/B and exon XL ~35 kb. Maternal (mat) and paternal (pat) *GNAS* products are illustrated above and below the gene structure, respectively, with splicing patterns indicated by broken lines. Filled boxes indicate untranslated sequences.

The human *GNAS* locus maps to the telomeric end of the long arm of chromosome 20 (20q13.2-20q13.3) [10-12], while its mouse ortholog is located in distal chromosome 2 [13, 14]. *GNAS* in humans and mice appear structurally and functionally similar to one and other. This locus has multiple promoters and differentially methylated regions (DMR) and gives rise to non-coding RNA molecules and transcripts that encode functional proteins (Fig. **1**). Furthermore, nearly all *GNAS* products show parent-of-origin specific expression. Among the multiple products derived from *GNAS*, the best characterized protein is the α-subunit of the heterotrimeric stimulatory G protein (Gsα), which has at least five variants that result from alternative pre-mRNA splicing. Derived from distinct promoters are at least four different additional *GNAS* products, including the neuroendocrine secretory protein 55 (NESP55), the extra-large variant of Gsα (XLαs), the A/B transcript, and the *GNAS* antisense transcript. Most of these additional transcripts also undergo alternative splicing and, thus, have different variants.

Gsα is a ubiquitous protein whose activity is essential for the cellular actions of many neurotransmitters, autocrine/paracrine factors, and hormones. As in α-subunits of other heterotrimeric G proteins, activation of Gsα by an agonist-occupied cell surface receptor results in a GDP-GTP exchange on Gsα, causing dissociation of the latter from Gβγ subunits and, thereby, allowing both Gsα and Gβγ to stimulate their respective effectors. GTP-bound, free Gsα can directly activate several different effectors, including Src tyrosine kinase [15] and certain Ca-channels [16, 17]. However,by far the most ubiquitous and the most extensively investigated effector molecule stimulated by Gsα is adenylyl cyclase, an integral membrane protein that catalyzes the synthesis of the ubiquitous second messenger cyclic AMP (cAMP), thereby triggering an intracellular signaling cascade that brings about an agonist- and cell-specific response. The activation of adenylyl cyclase and other effectors by Gsα is tightly regulated. The intrinsic GTP hydrolase (GTPase) activity of Gsα reverts the GTP-bound Gsα to its GDP-bound state and, thereby, results in the re-assembly of the G protein heterotrimer, which can no longer mediate effector stimulation. Mutagenesis experiments often lead to alteration in activity and/or subcellular distribution, indicating that changes are non-tolerable at many of the amino acid positions. Particularly, Arg^201^ and Gln^227^ are critical, since modifications of these residues, such as ADP-ribosylation of Arg^201^ that can be induced by cholera toxin or mutations at either residue, lead to inhibition of the GTPase activity and, therefore, render Gsα constitutively active [18-21]. The receptor and GTPase dependent activation cycle of Gsα, structural features of Gsα protein, and the roles of specific Gs effectors have been reviewed in more detail elsewhere [22, 23].

**Fig. (2):**
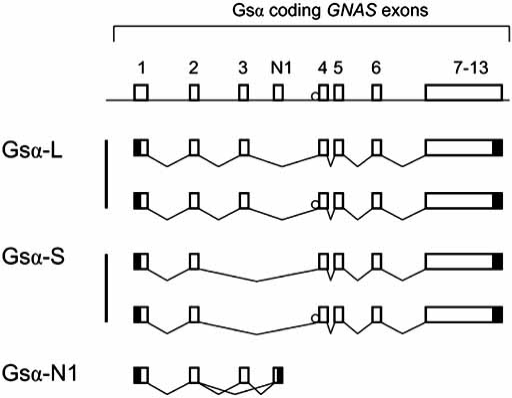
Splice variants of Gsα. Alternative splicing of exon 3 leads to Gsα-L and Gsα-S, each of which has subvariants due to alternative splicing of a serine codon at the start of exon 4. Gsα-N1 is formed through the use of an exon that comprises an in-frame termination codon and is located between exons 3 and 4. Boxes and connecting lines depict exons and introns, respectively. The alternatively spliced serine codon is depicted as a circle. Splicing patterns are indicated by broken lines. Filled boxes indicate 5’ and 3’ untranslated regions.

Gsα is encoded by 13 exons [24], but due to alternative pre-mRNA splicing, the Gsα transcript has several variants (Fig. **2**). The long and the short Gsα variants (Gsα-L and Gsα-S, respectively) differ from each other by the inclusion or exclusion of 45 nucleotides derived from exon 3 [24-26]. Nearly all tissues express both of these Gsα variants, which are typically detected as 52- and 45-kDa protein bands on Western blots. In addition, each Gsα form either includes or excludes a CAG tri-nucleotide (encoding serine) at the start of exon 4. Some investigations have revealed small, but potentially important, differences between the activities of Gsα-L and Gsα-S. For example, Gsα-L has been demonstrated to have a greater ability to transmit receptor signaling than Gsα-S when partially purified proteins from rabbit liver were examined [27], although the opposite was suggested in an assay system using cultured pancreatic islet cells [28]. Moreover, Gsα-L appears to release GDP ~2-fold faster than Gsα-S [29], and consistent with that finding, study of fusion proteins involving the β2-adrenergic receptor and either Gsα-L or Gsα-S has shown higher constitutive activity of the receptor when it is associated with Gsα-L [30]. In addition, differences in the subcellular trafficking of these two variants have been reported in response to activation by agonist, forskolin (a direct activator of adenylyl cyclase), or GTPγS (a stable GTP analog) [31-33]. Currently, it remains unclear whether these differences translate into biologically significant effects, such as divergence in the variety of effectors and/or the efficiency of effector activation. An important recent finding regarding the long and short Gsα forms is that, for the first time, an inactivating mutation in exon 3 has been identified in a patient with pseudohypoparathyroidism type-Ia [34], a disorder known to be caused by inactivating mutations in Gsα-coding *GNAS* exons (see below). The patient with the exon 3 mutation has an apparently mild form of this disorder, consistent with the disruption of only one of the two main Gsα variants, i.e. Gsα-L [34]. It is conceivable that, depending on the effector selectivity and relative expression levels of Gsα-L and Gsα-S in different tissues, this mutation impairs agonist responses in an effector- and tissue-specific manner. This possibility remains unexplored.

Alternative pre-mRNA splicing leads to another Gsα variant, termed Gsα-N1 (Fig. **2**). Identified first in the brain, Gsα-N1 is truncated in the C-terminus due to splicing of exon 3 (or exon 2 in the case of Gsα-S) onto a distinct exon containing an in-frame termination codon [35]. Exon N1 is located between exons 3 and 4. Gsα-N1 lacks most functional domains of Gsα, and therefore, it is probably unable to function in a way similar to the latter. Its cellular role and its biological significance remain unknown.

## TISSUE-SPECIFIC Gsα IMPRINTING

Several studies have demonstrated that the expression of Gsα in most fetal and adult tissues is biallelic, consistent with the absence of differential methylation at its promoter [36-39]. However, in some human and mouse tissues, Gsα expression is predominantly maternal, i.e. paternal Gsα expression is silenced. For example, in the renal cortex, mice with paternal disruption of *Gnas* exon 2 exhibit Gsα mRNA and protein levels that are similar to those seen in wild-type littermates, whereas mice with maternal disruption of *Gnas* exon 2 are nearly devoid of Gsα mRNA and protein [40], indicating that renal cortical Gsα expression is derived mostly from the maternal allele. This monoallelic, parent-of-origin specific Gsα expression occurs in a tissue-specific manner, as both mice with the maternal disruption and mice with the paternal disruption show Gsα levels in the renal medulla that are about 50% of the levels seen in wild-type littermates [40]. Similar findings have been reported recently in mice heterozygous for either paternal or maternal disruption of *Gnas* exon 1, and these studies additionally demonstrated that Gsα expression is also predominantly maternal in the thyroid [41]. Consistent with these findings, the active maternal Gsα promoter shows a greater ratio of tri- to dimethylated histone-3 Lys^4^ compared to the silenced paternal promoter in the proximal tubule, whereas the amount of methylated histones is similar in maternal and paternal Gsα promoters in liver, a tissue in which Gsα is biallelic [42].

Monoallelic, maternal Gsα expression has also been documented in human tissues through the analysis of a single nucleotide polymorphism located in exon 5. Thus, Gsα is predominantly maternal in thyroid, pituitary, and ovaries [43-45]. On the other hand, biallelic Gsα expression has been demonstrated in lymphocytes, adrenal, adipocytes, and bone cells [44, 46, 47]. The tissue-specific, imprinted expression of Gsα has important implications in the pathogenesis of diseases caused by mutations within *GNAS*, particularly pseudohypoparathyroidism (see below).

Several Gsα knockout mouse models have been generated in order to study the role of Gsα *in vivo*. The mouse models with universal Gsα disruption will be discussed below in the context of human diseases that are caused by heterozygous inactivating mutations within one of the 13 exons encoding this protein. In addition, Gsα has been conditionally ablated, through the use of the Cre-Flox technology, in specific tissues including liver, cartilage, bone, and glomerulus, confirming the absolute requirement of Gsα in the proper function of these tissues [48-51].

## NOVEL IMPRINTED GENE PRODUCTS OF THE *GNAS* LOCUS

Human and mouse studies over the past several years have identified novel promoters and exons within *GNAS*, thereby unveiling the previously unrecognized complexity of this gene locus. The sense strand comprises at least three promoters besides the promoter driving the expression of the aforementioned Gsα variants (Fig. **2**). In addition, there is at least one promoter that shows activity on the antisense strand. All of the recently described promoters are located within CpG islands that show differential, parent-of-origin specific methylation. Therefore, the *GNAS* locus harbors a total of four distinct DMRs. Consistent with the differential methylation of its promoter, each of the additional *GNAS* product shows an imprinted expression profile in nearly all tissues investigated in that regard.

### NESP55

The most upstream of the different *GNAS* promoters with respect to the promoter of Gsα drives the expression of NESP55, a neuroendocrine secretory protein with an apparent molecular mass of 55,000 (Fig. **1**). The NESP55 promoter is differentially methylated and active on the non-methylated maternal allele only [37, 52]. In humans, NESP55 protein is encoded by a single exon, while in mice, the ORF consists of two separate exons; however, in both species, these exons splice onto Gsα exons 2-13, which comprise the 3’ untranslated region (UTR) [37, 52]. The predicted molecular mass of NESP55 is 28 kDa, but it has the same electrophoretic mobility as a protein of 55 kDa [53]. NESP55 is a chromogranin-like protein expressed in neuroendocrine tissues, peripheral and central nervous system, and some endocrine tissues [53-56]. It is associated with the constitutive secretory pathway [57] and can be located in cholinergic, peptidergic, and adrenergic neurons [58]. In AtT20 cells, a corticotroph-like cell line, NESP55 has been shown to be exported out into the medium, a process which can be blocked markedly by lowering the temperature and modestly by treatment with a cAMP analog (8-bromo-cAMP) [59]. In different tissues, differential post-translational processing of NESP55 leads to smaller peptides, which appear to accumulate during antegrade transport of this protein along the axon [58]. One of the putative peptide products, Leu-Ser-Ala-Leu (LSAL), which may be produced by prohormone convertase cleavage, has been identified as an endogenous antagonist of the serotonergic 5-HT1B receptor subtype [53], although this possibility and its potential biological significance have yet to be investigated.

A knockout of the Nesp55 transcript has been generated in mice through introduction, by homologous recombination in ES cells, of a small deletion at the translation initiation site [60]. This mutation leads to a complete ablation of Nesp55 protein after maternal transmission, without affecting the expression of other *Gnas* transcripts. Nesp55 knockout mice appear to have no overt phenotypic abnormalities and are fertile. However, behavioral studies of these mice using several distinct tasks have revealed increased reactivity to novel environments, but these mice do not differ from wild-type mice in general locomotion and anxiety. Although the role of this protein at the molecular level remains to be explored, the findings in the Nesp55 deficient mice appear to be consistent with the localization of this protein in specific parts of the central nervous system, particularly noradrenergic locus coeruleus [61]. In humans, loss of NESP55 expression occurs as a result of a gain of methylation at its promoter (observed in some patients with pseudohypoparathyroidism type-Ib). This defect, however, does not appear to lead to an obvious phenotype, because no significant differences have been identified between patients with the gain of NESP55 methylation and those without this epigenetic alteration [62, 63].

### XLαs

Another promoter, located ~11 kb downstream of the NESP55 promoter, drives the expression of XLαs mRNA, which encodes a protein with partial identity to Gsα (Fig. **1**). As in NESP55, the XLαs transcript uses a novel first exon (exon XL) that splices onto exons 2-13 of Gsα [36, 52]. Unlike in the case of NESP55, however, the in-frame termination codon for the XLαs transcript is the same as for Gsα, making the encoded XLαs and Gsα proteins identical over a long C-terminal stretch [64]. Exon XL and the XLαs promoter are located in a CpG island methylated on the maternal allele [36, 52]. Consistent with this epigenetic mark, XLαs is derived exclusively from the paternal allele in all investigated tissues [36, 52, 65]; however, variable biallelic expression of XLαs has been recently demonstrated in clonal bone stromal cells [47]. Most abundant expression of XLαs is detected in neuroendocrine tissues, particularly pituitary, but the expression of its mRNA is readily detected by Northern blot or RT-PCR in various tissues, including brain, pancreas, heart, kidney, and adipose tissue [36, 64, 66, 67]. As demonstrated in the rat nervous system, XLαs expression is developmentally regulated [68].

Because XLαs shares significant amino acid identity with Gsα, it comprises most domains of the latter shown to be functionally important. Furthermore, the C-terminal end of the XL domain has significant homology to the exon 1 encoded portion of Gsα. Consistent with the high degree of overall similarity between XLαs and Gsα, various studies have demonstrated that XLαs is able to act in a manner similar to Gsα *in vitro*. First, XLαs show enhanced ADP ribosylation and an increased sucrose-density sedimentation rate upon addition of the Gβγ subunits, indicating that XLαs is able to form a heterotrimer [69]. Second, expression of an XLαs mutant carrying the homolog of the Gsα Gln^227^ mutation results in elevated cAMP formation, indicating that XLαs can stimulate adenylyl cyclase at least in the basal state [69]. Third, XLαs can mediate receptor-stimulated cAMP formation when overexpressed in opossum kidney cells that show endogenous Gsα expression [70] and in mouse embryonic fibroblasts that endogenously lack Gsα and XLαs due to homozygous disruption of *Gnas* exon 2 [71]. Finally, mutations that impair Gsα activity have similar effects on XLαs activity when introduced into the backbone of the latter [70]. Interestingly, however, beta-adrenergic stimulation seemingly fails to elicit cAMP generation in XLαs transfected S49 lymphoma cells that are also Gsα deficient (cyc^-^ clone) [69]. Furthermore, attempts to show coupling of XLαs to receptors have failed, using PC12 cells transfected with XLαs cDNA and pituitary membranes [69]. Taken together, these findings suggest that the Gsα-like activity of XLαs may be cell specific.

As illustrated in Fig. (**3A**), XLαs has several different variants. Similar to Gsα, it uses exon 3 alternatively, thus having at least a long and a short variant. In addition, analogous to Gsα-N1, a C-terminally truncated XLαs variant, termed XLαs-N1, also exists. Human XLαs-N1 appears to have a subvariant produced by inclusion of sequences from two additional exons, A20 and A21, located immediately following exon XL [36]. A similar subvariant has also been demonstrated for rat XLαs-N1 [66]. The introduction of these additional sequences into XLαs-N1 results in a frame-shift, leading to a putative protein with partial identity to XLαs but not to Gsα. Functional significance of XLαs-N1 and its variants remains unknown.

**Fig. (3):**
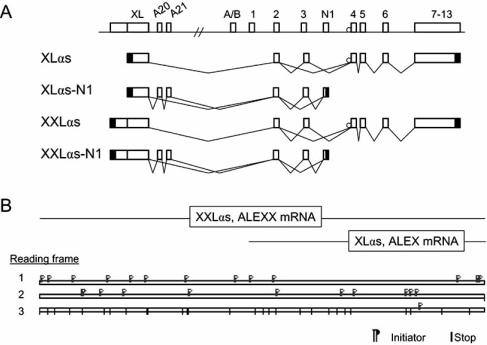
XLαs and its multiple variants. XLαs is derived from a promoter upstream of that which drives the expression of Gsα . Panel A. Alternative splicing leads to different XLαs variants that are analogues to Gsα variants. In addition, an N-terminally extended XLαs variant, termed XXLαs, is made, representing the 5’ extension of the ORF. For simplicity, GNAS exons located upstream of exon XL are not shown. Panel B. XLαs and XXLαs mRNA include two ORFs each. The second ORF leads to ALEX or ALEXX, respectively.

There is evidence for the existence of a long XLαs transcript, termed XXLαs, which extends in the 5’ direction (Fig. **3A**). The XXLαs transcript is supported by Est databases and a single study that could amplify portions of the XXLαs transcript by RT-PCR [72]. A long XLαs transcript has also been revealed by Northern blot in mouse heart and adipose tissue [67]. In fact, based on existing data, the translation initiation codon for XLαs does not match with that corresponding to the longest open reading frame (ORF), making it likely that an N-terminally extended XLαs variant is expressed at least in some tissues. Est databases also suggest the existence of transcripts that correspond to XXLαs-N1, but neither the role of XXLαs nor the role of XXLαs-N1 is currently known. Interestingly, however, unlike the XL domain in XLαs and XLαs-N1-- the portion encoded by exon XL -- the N-terminally extended part in XXLαs and XXLαs-N1 is highly conserved among mammals, suggesting that it may represent a functionally significant domain.

The XLαs mRNA has a second ORF that encodes another protein termed ALEX (for alternative protein encoded by XLαs mRNA), which uses a termination codon located within exon XL and is, therefore, smaller than XLαs (Fig. **3B**). *In vitro* studies have shown that ALEX, which has a predicted molecular mass of 38 kDa, can interact with XLαs [73, 74]. An insertion polymorphism within XLαs mRNA, affecting both proteins, appears to impair this interaction, a finding that has been offered as an explanation as to why some individuals carrying this insertion polymorphism show elevated agonist-induced cAMP accumulation in platelets [74]. However, this increased agonist responsiveness could also result from elevated Gsα levels, which was demonstrated in several individuals who carried the same insertion polymorphism and had increased bleeding tendency [75]. Hence, the significance of XLαs-ALEX interaction on XLαs or Gsα function remains to be clarified. Of note, this alternative ORF also extends in to the XXLαs mRNA, generating a putative N-terminally extended ALEX variant, termed ALEXX [72]; however, there are currently no available experimental data to suggest that this protein actually exists.

Exon XL has been disrupted in mice through introduction of a small deletion into the beginning part of both XLαs and ALEX ORF; this disruption appears to preserve the imprinted expression of other *Gnas* transcripts [67]. The mice with paternal inheritance of the disrupted XLαs allele (*Gnas*xl knockout mice) lack XLαs expression, consistent with the exclusive paternal expression of the XLαs transcript. The *Gnas*xl knockout pups show defective suckling, hypoglycemia, growth retardation, and early postnatal mortality. Despite having hypoglycemia, the *Gnas*xl knockout mice show reduced glucagon levels and inappropriately normal serum catecholamine and cortisol levels, suggesting a possible impairment of the physiological responses to hypoglycemia. When crossed into the CD1 background, about 20% of the *Gnas*xl knockout animals survive with an apparently normal life-span. However, these mice display increased glucose tolerance and insulin sensitivity, as well as a hypermetabolic state associated with reduced adiposity and hypolipidemia [67]. In addition, circulating levels of norepinephrine is elevated in these animals, indicating increased sympathetic nervous system activity, which explains, at least in part, the defects in the energy metabolism [76]. On the other hand, adipocyte-autonomous factors appear less likely to be involved in these phenotypes, because XLαs is not expressed in the adipocytes of adult wild-type mice [76].

The genetic alteration in the *Gnas*xl knockout mice is predicted to disrupt not only XLαs but also XLαs-N1, ALEX, XXLαs, XXLαs-N1 and ALEXX. It thus appears unlikely that the phenotype of *Gnas*xl knockout mice is due solely to XLαs deficiency. Nonetheless, a similar phenotype is present in mice with paternal *Gnas* exon 2 disruption [77-79] and in mice with a paternally inherited missense mutation in exon 6 [80, 81], thereby ruling out the involvement of ALEX, XLαs-N1, and XXLαs-N1 deficiency in the phenotypes of the *Gnas*xl knockout mice. Thus, it is more likely that the deficiency of XLαs and/or XXLαs underlies the findings observed in the latter mouse model, although this conclusion needs to be verified through further investigation. It is important to note that the phenotype of the *Gnas*xl knockout mice differs, by and large, from the phenotype of mice heterozygous for disruption of *Gnas* exon 1, in which Gsα, but not XLαs, is ablated [41, 82]. In fact, *Gnas*xl knockout mice demonstrate slightly elevated basal and isoproterenol stimulated cAMP levels in brown adipose tissue at birth [67]. These findings suggest that XLαs has a role distinct from the role of Gsα in mammals, and that it may oppose, at least in certain tissues, the actions of Gsα.

Findings that are reminiscent of those observed in *Gnas*xl knockout mice and the mice with paternal *Gnas* exon 2 disruption have been reported in two unrelated children with large paternal deletions of chromosome 20q13.3 that comprise the *GNAS* locus [83]. These findings included perinatal growth retardation, intractable feeding difficulties, and loss of subcutaneous adiposity, as well as dysmorphic facial features. Because the deletion involved, in each case, the entire *GNAS* locus, it is possible that the phenotype reflects, at least partially, the deficiency of Gsα and/or the other paternally expressed *GNAS* products (see below).

### The A/B Transcript

About 2.5 kb upstream of the Gsα promoter lies another promoter that drives the nearly ubiquitous expression of another transcript termed A/B (also termed 1A and 1’) [84-86]. The A/B promoter, despite being in close proximity to the Gsα promoter, reside within a DMR, where the maternal allele is methylated and repressed, while the paternal allele is non-methylated and active (Fig. **2**). The first exon of the A/B transcript, as in NESP55 and XLαs, splices onto Gsα exons 2-13. However, exon A/B does not contain an in-frame translation initiation codon, and as supported by evidence from *in vitro* translation assays [86], translation can be initiated by an in-frame AUG located in exon 2, leading, presumably, to a variant of Gsα that has a truncated N-terminus. Transfection of COS cells with A/B cDNA results in the expression of a protein that localizes to the plasma membrane [86]. Based on these features, it is conceivable that the A/B protein interacts with adenylyl cyclase and may, therefore, exert a dominant negative effect on Gsα actions. This possibility remains to be investigated.

On the other hand, it appears more likely that the A/B transcript is non-coding, as the existence of an endogenous A/B protein is not supported by experimental evidence. Furthermore, similar to other non-coding RNA molecules in the genome, the A/B transcript and/or the exon A/B DMR has an important role in regulating gene expression from *GNAS*. Unlike the DMRs comprising the promoters of NESP55 and XLαs, the exon A/B DMR has been shown to be a germ-line imprint mark [85]. It has also been shown that this DMR is associated with allele-specific differences in histone modifications consistent with an active paternal (histone acetylation and histone-3 Lys^4^ methylation) and an inactive maternal (histone-3 Lys^9^ metyhlation) promoter [42, 87]. In addition, paternal ablation of the A/B DMR results in derepression of the Gsα transcript in those tissues where paternal Gsα expression is normally silenced, such as the renal cortex [81, 88]. Thus, it appears that the non-methylated A/B DMR and/or active A/B transcription is necessary for the tissue-specific paternal silencing of Gsα. Consistent with this finding, maternal exon A/B is unmethylated and the maternal A/B promoter is derepressed in patients with pseudohypoparathyroidism type-Ib [63, 89, 90], who are thought to have a lack of Gsα expression in the proximal tubule due to silencing of the maternal Gsα promoter (in addition to the silencing of the paternal Gsα promoter that occurs normally in the same tissue; see below for further discussion).

The mechanisms underlying the role of A/B in the tissue-specific silencing of paternal Gsα expression are not well understood. Considering that the A/B transcription takes place more broadly than does paternal Gsα silencing, a mechanism that involves simple competition between the promoters of these two transcripts is unlikely. On the other hand, a plausible hypothesis involves a mechanism whereby the paternal A/B DMR binds either a repressor that directly silences the Gsα promoter or an insulator that prevents the effects of an upstream enhancer on the Gsα promoter. A mechanism similar to the latter has been demonstrated for the Igf2-H19 locus, in which methylation-sensitive binding of CTCF hinders Igf2 enhancer activity [91, 92]. To address these possibilities, it may be necessary to generate and study additional mouse models in which A/B transcript is disrupted without the deletion of its promoter region.

### The *GNAS* Antisense Transcript

A promoter located immediately upstream of the XLαs promoter drives expression of an antisense transcript, which extends past the exon(s) encoding NESP55 [93, 94] (Fig. **2**). In mice, the mature antisense transcript (termed Nespas or *Gnas*as) encompasses both Nesp55 exons 1 and 2 and the intervening intron [94], while in humans, it has no overlap with exon NESP55 [93]. There are at least five distinct exons that form the human *GNAS* antisense transcript, but alternative splicing results in at least six variants. The largest open reading frame is predicted to encode a polypeptide of 97 amino acids that share no homology to known proteins [93]. Taken together these features suggest that the antisense transcript is non-coding. Indeed, similar to the promoter of A/B, the promoter of the *GNAS* antisense promoter resides in a DMR and is active exclusively on the paternal allele [65, 93, 94]; however, the antisense transcript shows biallelic expression in the adrenal and testes [65]. Consistent with allele-specific expression, the paternal antisense promoter is associated with acetylated histone and histone-3 Lys^4^ methylation, while the maternal promoter lacks histone acetylation and carries histone-3 Lys^9^ methylation [87]. Furthermore, studies in mice have shown that the maternal methylation at the antisense promoter is present even in the oocytes and, therefore, represents a germ-line imprint mark [95].

Paternal deletion of the antisense promoter in mice results in derepression of Nesp55 *in cis*, thereby leading to biallelic expression of the latter [96]. This finding indicates that at least one of the roles of the antisense transcript is to silence the paternal Nesp55 promoter. However, the effect of the deletion of the antisense promoter is not limited to Nesp55 expression. First, the deleted region evidently comprises an enhancer of the XLαs promoter, resulting in a dramatic reduction in XLαs expression and, thus, a phenotype similar to that observed in *Gnas*xl knockout mice. Second, mice with the paternal deletion exhibit a modest decrease in A/B expression combined with an increase in the methylation of the A/B promoter. Third, in those tissues where Gsα is normally silenced from the paternal allele, the diminished A/B expression is associated with an increase in Gsα expression *in cis*, i.e. the tissue specific Gsα imprinting is relaxed. These findings clearly demonstrate the importance of the antisense transcript in the regulation of imprinted gene expression from the *GNAS* locus. Nonetheless, what causes the reduction of paternal A/B expression remains unclear. Is it the derepression of Nesp55, the reduction of XLαs expression, or the deletion of the genomic region containing the antisense promoter?

## HUMAN DISEASES ASSOCIATED WITH *GNAS* MUTATIONS

Consistent with the pivotal role of Gsα in multiple biological responses, mutations that affect the activity or expression of Gsα lead to human disease. However, there are no disorders caused by inactivation of both Gsα alleles, i.e. homozygous inactivating Gsα mutations, and this is consistent with the early embryonic lethality observed in mice with homozygous disruption of either *Gnas* exon 2 or *Gnas* exon 1 [40, 41, 82]. Thus, complete loss of Gsα activity is not compatible with life. Moreover, there are mutations that cause constitutive Gsα activity, but these are virtually never inherited and are of somatic origin, indicating that universal Gsα overactivity is embryonic lethal, as well.

### Endocrine Adenomas and other Tumors

Many hormones bind Gsα-coupled receptors in order to activate their endocrine glands for proliferation, differentiation, and hormone secretion. Accordingly, mutations that cause constitutive Gsα activity are found in various functionally active endocrine adenomas, including those that originate from pituitary somatotrophs. In about 40% of patients with growth hormone secreting pituitary adenomas, constitutively activating Gsα missense mutations at either Arg^201^ or Gln^227^ have been identified in DNA from the tumor tissue, but not in DNA from peripheral blood [20, 97]. Since these mutant Gsα forms are identified in tumors and are present in one of the Gsα alleles only, they are referred to as the *gsp* oncogene [20, 97]. Other endocrine tumors also bare the *gsp* oncogene, including corticotroph, thyroid, parathyroid, and adrenocortical tumors, but their frequency in the patient population appears to be low based on many studies (reviewed in [98]). Some studies have recently identified the *gsp* oncogene in ovarian granulosa cell tumors and testicular stromal Leydig cell tumors as a possible cause of tumorigenesis and as a possible prognostic marker [99, 100]. In a more recent study, 5 of 30 patients with clear cell renal carcinoma have been shown to carry constitutively activating Gsα mutations in the tumor tissue [101]. Hence, the *gsp* oncogene can be present in a wide variety of tumors that mostly, but not exclusively, involve classic endocrine tissues. Data from transgenic mouse models and cell culture assays have shown that constitutive Gsα activity can lead to hyperplasia and increased hormone secretion in endocrine cells [102, 103]. However, although a recent study implicates sustained activation of extracellular signal-regulated kinase in increased hormone secretion [104], the signaling pathways downstream of the *gsp* oncogene currently remain incompletely understood.

### McCune-Albright Syndrome

This syndrome, independently described by McCune [105] and Albright *et al*. [106], is characterized by a triad of sexual precocity, fibrous dysplasia of bone, and hyperpigmented skin lesions termed café-au-lait spots. Patients with the McCune-Albright syndrome (MAS) are mosaic for constitutively activating Gsα mutations that occur during early embryonic development [107-109]. This is consistent with the observation that MAS occurs sporadically and is never transmitted to the next generation. All identified mutations are at residue Arg^201^ (Cys or His), suggesting that changes in residue Gln^227^ may result in higher constitutive activity and are, therefore, less viable. Because of the mosaicism, patients with MAS show significant variation in their clinical presentation. In general, the abnormalities involve bone, skin, and endocrine organs. Fibrous dysplastic bone lesions are usually found in multiple bones. It is important to note that some patients with the constitutively activating Gsα mutations present with fibrous dysplasia alone, affecting either a single bone or multiple bones. In patients with isolated fibrous dysplasia, histological changes appear to be indistinguishable form those seen in the context of MAS [110, 111]. As in patients with MAS, nearly all isolated cases of fibrous dysplasia are associated with *GNAS* mutations at Arg^201^; however, a study using a mutation-specific restriction enzyme digest assay has recently identified three Gln^227^ (to Leu) mutations among a total of 56 samples [112]. The skin lesions, which typically have irregular borders, can be single or multiple light brown hyperpigmented areas arranged in segmental patterns that follow the developmental lines of Blaschko [106, 109]. Endocrine abnormalities, in addition to precocious puberty, can be summarized as hyperplasia and increased function of many different glands, including thyroid, adrenal, and pituitary [113-115]. Some patients with MAS also exhibit urinary phosphate wasting, hypophosphatemia, and bone mineralization defects observed as rickets or osteomalacia [113, 114]. While the latter findings are consistent with the role of Gsα in mediating the phosphaturic actions of parathyroid hormone in the renal proximal tubule, recent data shows that serum phosphate in MAS patients is negatively correlated with the level of fibroblast growth factor-23 [116-118], suggesting that the elevation of this phosphaturic factor is responsible, at least partly, for the hypophosphatemia observed in these patients. In addition to the various endocrine defects discussed above, there are rare reports of non-endocrine abnormalities associated with MAS, including liver and cardiac abnormalities, and neurological defects. The endocrine and non-endocrine findings in MAS have been reviewed elsewhere in greater detail [98].

### Pseudohypoparathyroidism Type-Ia, Pseudopseudohypoparathyroidism, and Progressive Osseous Heteroplasia

Fist described by Albright and colleagues [119], Pseudohypoparathyroidism (PHP) refers to end-organ resistance to multiple hormones that primarily involves the actions of parathyroid hormone. PTH exerts its actions in bone and kidney through the PTH/parathyroid hormone-related peptide receptor (PTHR1), which couples to Gs and, less effectively, to Gq [120, 121]. PTH increases bone turnover, leading to mobilization of calcium and phosphate from bone [122, 123]. In the proximal renal tubule, it induces the synthesis of 1,25-dihydroxyvitamin D and inhibits phosphate reabsorption from the glomerular filtrate, while in the distal renal tubule, it enhances the absorption of calcium mediated *via *transcellular mechanisms. Exogenous administration of biologically active PTH, used previously as a diagnostic test [124], results in a blunted excretion of urinary phosphate in both PHP type-I and PHP type-II, but this defect is accompanied by blunted nephrogenous cAMP production in PHP type-I only; the PTH-induced generation of nephrogenous cAMP is normal in PHP type-II [119, 125]. Clinically, PHP-I is far more frequent than PHP-II, for which underlying molecular defects are not well understood. On the other hand, significant advances have been recently made regarding the molecular pathology underlying PHP-I.

Some patients with PHP-I display distinctive physical features collectively termed Albright’s hereditary osteodystrophy [119]. These features include obesity, short stature, ectopic ossification, brachydactyly, and mild mental retardation, although there is significant patient-to-patient variation in the range and severity of these features. The presence of both PTH-resistance and AHO defines patients with PHP type-Ia. Gsα mRNA and protein levels are reduced to half in easily accessible tissues from these patients [126-128]. This defect results from heterozygous inactivating mutations within one of the thirteen Gsα coding *GNAS* exons [129, 130]. Scattered throughout the gene, various different types of mutations, such as insertions, deletions, and missense and nonsense changes, have been identified, which is consistent with their inactivating nature. An extensive list of the mutations associated with this disorder can be found under OMIM entry #139320 at http://www.ncbi.nlm.nih.gov.

Because Gsα is not exclusive for PTH signaling, PHP-Ia patients also show resistance to some other hormones whose actions depend on Gsα signaling, including thyroid stimulating hormone (TSH), gonadotropins, and growth hormone releasing hormone (GHRH) [131-135]. It is worth noting that not all hormone actions that rely on Gsα are impaired in PHP-Ia. For example, there is no resistance to vasopressin [136, 137] or to hormones in the hypothalomo-pituitary-adrenal axis [134, 136, 138].

Inactivating Gsα mutations found in PHP-Ia patients are also present in patients who lack hormone resistance but present with AHO features, a condition referred to as pseudopseudohypoparathyroidism (PPHP) [139]. Mutations in patients with PPHP are often identical to those in PHP-Ia patients. In fact, both disorders typically co-exist in the same kindred [129, 140], with the gender of the affected parent determining whether the offspring will have PHP-Ia or PPHP: maternal inheritance leads to PHP-Ia, whereas paternal inheritance leads to PPHP [141, 142]. Thus, hormone resistance (PHP-Ia) is inherited only from female obligate carriers, a mode of inheritance that is consistent with the predominantly maternal expression of Gsα in certain tissues. It is important to note that the repertoire of hormone resistance in PHP-Ia correlates well with the tissues in which maternal, monoallelic Gsα expression takes place, underscoring the significance of Gsα imprinting in the pathogenesis of PHP-Ia.

AHO features are present in both PHP-Ia and PPHP patients, and therefore, the molecular mechanisms underlying AHO presumably entail Gsα haploinsufficiency rather than imprinting. Gsα haploinsufficiency has been demonstrated in the growth plate of mice chimeric for wild-type cells and cells heterozygous for disruption of either the maternal or the paternal *Gnas* exon 2 [143]. In the chimeric setting, the mutant chondrocytes undergo hypertrophic differentiation sooner than wild-type chondrocytes, and because this finding is qualitatively similar to (albeit far less severe than) that observed in chondrocytes with homozygous Gsα ablation under the same conditions, it indicates Gsα haploinsufficiency. While this study strongly suggests that the short stature and the brachydactyly seen in patients with AHO reflect, at least in part, Gsα haploinsufficiency in the growth plate, some features of AHO may still involve Gsα imprinting in the pathogenesis. For example, a recent study has clearly shown that obesity is more prominent in PHP-Ia patients than PPHP patients [144]. Thus, it appears that Gsα may be imprinted in more tissues than currently recognized, such as in parts of the brain that controls satiety and body weight. It is also possible that disruption of other imprinted *GNAS* gene products contribute to the pathogenesis of AHO. Supporting this hypothesis, chondrocytes with paternal *Gnas* exon 2 disruption exhibit a slightly, but significantly, higher degree of Gsα haploinsufficiency than chondrocytes with maternal *Gnas* exon 2 disruption [143]. More detailed characterization of the different AHO features between patients with paternally and maternally inherited Gsα mutations are likely to provide further insights into the understanding of the mechanisms underlying AHO.

Progressive osseous heteroplasia (POH) describes a severe, debilitating disease characterized by ectopic intramembranous bone formation that affects not only the subcutis, but also the skeletal muscle and the deep connective tissue [145]. Heterozygous inactivation mutations within the Gsα coding *GNAS* exons have also been identified in patients with POH. In fact, some of those mutations are identical to those found in patients with PHP-Ia or PPHP [145-147]. It is therefore possible that POH is an extreme manifestation of the ectopic bone formation of AHO that normally involves the subcutaneous tissue. However, POH is rarely accompanied with any AHO features or hormone resistance [146, 148]. Furthermore, in many kindreds, it has been shown that the disease develops only after paternal inheritance [147], suggesting that genomic imprinting also plays a role in the pathogenesis of POH. Because this disorder is paternally inherited, deficiency of *GNAS* products that show paternal specific expression and share exons with Gsα, such as XLαs, could contribute to ectopic bone formation. Consistent with this hypothesis, no mutations in exon 1 has thus far been reported in cases with isolated POH [147, 149, 150]. However, the roles of XLαs and other imprinted *GNAS* products in POH remain currently undefined.

### PHP Type-Ib

Some patients with PTH-resistance lack AHO and any additional hormone resistance, defining the typical features of PHP type-Ib (PHP-Ib). Recent studies have demonstrated that some PHP-Ib patients also have mild TSH resistance in addition to PTH resistance [89, 90, 151]. Furthermore, a single study described several patients who showed both the epigenetic defects characteristic of PHP-Ib (see below) and mild features of AHO [152], suggesting more variation in the phenotype. Unlike in PHP-Ia, Gsα activity is typically normal in easily accessible cells from these patients, thus excluding mutations within Gsα coding *GNAS* exons [131, 153]. Nonetheless, in three related patients with an apparent diagnosis of PHP-Ib an in-frame, tri-nucleotide deletion has been identified within exon 13 [154]. Expressed in HEK293 cells, an embryonic kidney derived cell line, this Gsα mutant was shown to affect the signaling of PTH, but not of TSH, LH, or isoproterenol, thus explaining the isolated PTH resistance. The selective effect of this mutation on PTH signaling, however, could not be verified in a subsequent study [70], and it is possible that the discrepancy between the two studies stems from the use of different cell types and/or assays; the second study used mouse embryonic fibroblasts *null* for endogenous Gsα [70, 71]. Alternatively, the three patients may have PHP-Ia, consistent with the observation that two of them exhibited advanced bone age [154], which is a typical sign of AHO. Since the urinary cAMP response to exogenously administered PTH is blunted in PHP-Ib patients [131], defects in the gene encoding PTHR1 seemed like a good candidate at the time. However, several studies have ruled out this possibility [153, 155-157].

**Fig. (4):**
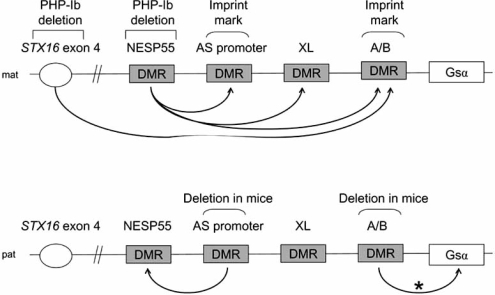
Regulation of gene expression from the *GNAS* locus. The maternal *GNAS* allele comprises two germ-line imprint marks, one at the AS promoter and the other at exon A/B. Ablation of the non-methylated, paternal AS promoter causes derepression of NESP55, and ablation of the non-methylated, paternal exon A/B leads to derepression of Gsα; the latter occurs in tissues where the latter is normally silenced from the paternal allele (*). Most familial PHP-Ib cases exhibit isolated loss of *GNAS* exon A/B imprinting. Deletions identified in those cases point to a region within the *STX16* locus, probably around exon 4, as comprising a cis-acting long-range regulatory element that is necessary for the establishment of the maternal exon A/B imprint. All *GNAS* maternal imprints are lost in most sporadic and some familial PHP-Ib cases. Deletions identified in the latter suggest that the NESP55 DMR, which contains not only exon NESP55 but also exons 3 and 4 of the AS transcript, comprises a cis-acting element that controls the imprinting of the entire maternal *GNAS* allele. Arrows indicate the regulatory effects revealed by mutations in PHP-Ib and the study of knockout mouse models. mat, maternal; pat, paternal.

PHP-Ib is often sporadic, but a number of familial cases have also been described. A careful analysis of these pedigrees has revealed that the PTH resistance in PHP-Ib develops only if the genetic defect is inherited from a female carrier [158], i.e. the mode of inheritance is identical to that observed for hormone resistance in PHP-Ia. Genetic linkage studies using some of these kindreds have mapped the genetic defect to a region of chromosome 20q that comprises the *GNAS* locus [158, 159]. Moreover, most PHP-Ib patients exhibit *GNAS* imprinting defects [63, 89]. Although these defects involve multiple *GNAS* DMRs in some cases, the most consistent defect is a loss of imprinting at the A/B DMR, i.e. loss of methylation at the A/B promoter and exon combined with biallelic A/B expression, which appears to be an isolated defect in most familial PHP-Ib cases [89, 160]. Based on these findings, the genetic mutation responsible for PHP-Ib is thought to disrupt an imprinting regulatory element of *GNAS*. The most frequent mutation, identified thus far in more than 30 unrelated kindreds, is a unique 3-kb microdeletion located about 220 kb upstream of exon A/B [160-163] (Fig. **4**). Flanked by two 391-bp repeats, the 3-kb microdeletion removes exons 4-6 of *STX16*, the gene encoding syntaxin-16. The second mutation is a 4.4-kb microdeletion that overlaps with the former and removes *STX16* exons 2-4; this mutation, unlike the 3-kb microdeletion, has been found in only one kindred thus far [164]. These mutations cause disease only after maternal inheritance, and each affected individual carrying either of these mutations displays an isolated loss of exon A/B imprinting, thereby indicating that the mutations disrupt a cis-acting element that controls imprinting at the exon A/B DMR [160, 164]. This element may lie within the 1.3-kb region where the two deletions overlap. The overlapping region comprises exon 4, which is evolutionarily conserved and lies within a small CpG-rich region that lacks differential methylation [160]. It is also possible that the identified deletions independently disrupt a large control element that spans *STX16*. On the other hand, disruption of *STX16* is not considered to be involved in the pathogenesis of PHP-Ib, because there is no evidence that this gene is imprinted [160, 164].

All sporadic and some familial PHP-Ib cases show epigenetic defects at one or more *GNAS* DMRs in addition to the exon A/B DMR [63, 89]. These defects often consist of a loss of imprinting at exon A/B, exon XL, and the promoter of the antisense transcript and a gain of imprinting at exon NESP55. Two unrelated familial cases with such *GNAS* imprinting abnormalities have been shown to carry maternally inherited deletions of the entire NESP55 DMR including exons 3 and 4 of the antisense transcript [165], revealing the putative location of another control element required for the imprinting of the entire maternal *GNAS* allele (Fig. **4**). The presence of similarly large deletions at the NESP55 DMR has been excluded in a number of sporadic PHP-Ib cases [165].

The broad *GNAS* epigenetic defects seen in the sporadic cases are often such that the maternal allele has attained a paternal epigenotype. Accordingly, a sporadic PHP-Ib case has been shown to have paternal uniparental isodisomy of the entire chromosome 20q (patUPD20q) [90]. Leading to the diagnosis were PTH-resistance and mild TSH resistance in the absence of typical AHO findings, although the patient had additional abnormalities, including developmental delay and craniosynostosis, which may have resulted from either disrupted expression of other imprinted genes or from unmasking of recessive defects present on paternal chromosome 20q. Hence, patUPD20q is a cause of sporadic PHP-Ib, and it is possible that interstitial paternal UPDs in this region could lead to PHP-Ib as a more common cause of this disorder in the sporadic cases. It has also been suggested that some cases of sporadic PHP-Ib is caused by stochastic defects in the imprinting process [162]. In either case, the offspring of affected females would be predicted to have normal *GNAS* imprinting and normal proximal tubular PTH responsiveness even if they inherit the disease-associate allele.

Sporadic PHP-Ib cases typically differ from familial PHP-Ib cases in the nature of the *GNAS* epigenetic defects they exhibit, yet evaluation of many cases with these PHP forms indicates that the divergence in the epigenetic features do not directly translate into differences in clinical presentation. The age of onset and the severity of hypocalcemia and hyperphosphatemia that result from PTH resistance appear to be similar in sporadic and familial cases [62]. As explained above, Gsα expression is also predominantly maternal in the pituitary somatotrophs [43]; however, growth hormone deficiency and short stature are not typical clinical features of PHP-Ib. It is possible that the epigenetic defects do not affect Gsα expression in this tissue, thereby allowing unimpaired GHRH signaling. This could suggest that the mechanisms underlying Gsα imprinting are different between the pituitary and the proximal tubule. Alternatively, GHRH resistance may be present in patients with PHP-Ib, but it may be too mild to become clinically manifest. This is similar to the TSH resistance in PHP-Ib, which can be absent in many PHP-Ib patients, is milder than in PHP-Ia, and can be accounted for by the partial imprinting of Gsα in the thyroid [151]. Mild short-stature has been recently reported in some patients who show PTH-resistance and *GNAS* imprinting defects [152], consistent with GHRH resistance and resultant growth hormone deficiency. Nevertheless, it remains unknown whether patients with PHP-Ib have reduced Gsα expression in the pituitary and whether they display GHRH resistance.

The common defect in nearly all PHP-Ib cases is the loss of *GNAS* exon A/B imprinting. In fact, this epigenetic defect is necessary for the development of PTH resistance, as documented in a PHP-Ib kindred in whom some individuals lacked both loss of A/B imprinting and PTH resistance despite maternally inheriting the disease-associated haplotype on 20q [166]. The loss of A/B imprinting on the maternal allele is predicted to silence, *in cis*, Gsα transcription in the proximal tubule. Therefore, methylation of exon A/B DMR and/or the repression of A/B transcription appear to be required for maternal Gsα expression in this and, probably, other tissues, such as the thyroid gland. Supporting this notion, ablation of the paternal exon A/B region derepresses Gsα *in cis* in tissues where this signaling protein is paternally silenced [81, 88] and, furthermore, rescues the PTH resistance phenotype observed in mice with a point mutation in maternal *Gnas* exon 6 [80, 81].

## ANIMAL MODELS OF THE *GNAS*-RELATED DISEASES

### PHP-Ia/PPHP

Before the evidence that several different *GNAS* transcripts, in addition to Gsα, utilize exon 2, a mouse model of PHP-Ia was generated by targeted disruption of this exon [40]. Homozygous disruption of exon 2 results in early embryonic lethality. Furthermore, paternal heterozygous disruption leads to lethality within the first 24 h after birth, and maternal heterozygous disruption results in death within the first three weeks. Surviving animals are fertile and appear to have normal life spans. As explained above, mice with paternal disruption of *Gnas* exon 2 have severe defects that are similar to those seen in mice with paternal disruption of *Gnas*xl [67], including reduced adiposity, which is not seen in patients with paternally inherited inactivating Gsα mutations (PPHP); these patients are typically overweight. On the other hand, both the maternal and the paternal *Gnas* exon 2 knockout mice appear smaller than their littermates, reminiscent of the short stature observed in patients with AHO. Furthermore, the exon 2 knockout mice prove to be a good model of PTH resistance. Reflecting the tissue-specific silencing of paternal Gsα in the proximal tubule, PTH-resistance, characterized by hypocalcemia, hyperphosphatemia, and elevated serum PTH, is present only after maternal inheritance of the disrupted allele [40]. In addition, PTH stimulation of proximal tubule extracts from mice with the maternal disruption, but not with the paternal disruption, fail to increase cAMP levels. Similar to the observations in PHP-Ia patients, the urine concentrating ability of the kidney in response to vasopressin is unimpaired in both the maternal and the paternal knockouts. Hence, although the findings resulted from the disruption of exon 2 are rather complicated due to the use of this exon by not only Gsα but also several other *GNAS* products (particularly XLαs), this animal model is able to phenocopy PHP-Ia to a significant extent.

Chen *et al. *[82] and Germain-Lee *et al. *[41] have independently generated another model of PHP-Ia through targeted disruption of *Gnas* exon 1. In addition to early embryonic lethality observed upon homozygous inheritance of exon 1 disruption, even the heterozygous disruption is associated with some pre-weaning mortality regardless of the parental origin. While this finding is not observed in families with PHP-Ia/PPHP, the overall phenotype of mice heterozygous for maternal disruption of *Gnas* exon 1 resembles that of PHP-Ia remarkably. Mice with maternal disruption of exon 1 develop biochemical features consistent with PTH resistance [41]. In humans with inactivating Gsα mutations it is accepted that obesity develops regardless of the parent-of-origin of the introduced mutation, but recent evidence indicates that patients with PHP-Ia exhibit more prominent obesity than patients with PPHP [144]. Consistent with this recent observation, mice with the maternal *Gnas* exon 1 disruption is more obese than the paternal disruption [82]. In contrast to these similarities between the mouse models of exon 1 ablation and PHP-Ia/PPHP, the serum PTH level seems to be moderately elevated in mice with paternal *Gnas* exon 1 ablation, suggesting the presence of PTH-resistance [41]. This finding is not observed in mice with paternal ablation of exon 2 and may therefore reflect the preservation of *Gnas* transcripts that use exon 2, such as XLαs. Although this conclusion is contrary to the evidence that XLαs can mimic Gsα [70, 71, 143], it correlates well with the notion that Gsα and XLαs, which are oppositely imprinted in certain tissues, mediate opposing actions [67].

### PHP-Ib

Since a 3-kb deletion removing exons 4-6 of the *STX16* locus has been identified in numerous familial PHP-Ib cases, and since this locus, with its exon-intron structure and its proximity to the *GNAS* locus, is similar in mice and humans, an attempt at generating a PHP-Ib mouse model has been made through targeted deletion of *Stx16* exons 4-6 [167]. Both heterozygous and homozygous mice with this deletion lack gross abnormalities, are fertile, and appear to have a normal life span. More important, regardless of the parental origin of the deletion, the mutant mice fail to phenocopy the epigenetic abnormalities found in patients with familial PHP-Ib. In fact, neither any other *Gnas* methylation defects nor any biochemical features that would suggest PTH resistance could be observed in these animals. Thus, the nearly exact change in the mouse *Stx16* gene is not sufficient to disrupt *Gnas* imprinting and to result in PTH resistance, ruling out a disrupted *STX16* mRNA and/or protein as the molecular cause of familial PHP-Ib. This conclusion, which is consistent with the evidence that the *STX16* locus is not imprinted, supports the hypothesis that the deletions in this gene disrupt a cis-acting long-range regulatory element required for the proper imprinting of *GNAS* exon A/B. It appears that this putative control element is not located within *Stx16* exons 4-6 in mice.

## SUMMARY AND CONCLUSION 

*GNAS* is a complex locus located on the telomeric end of chromosome 20q. At least five distinct promoters reside in this locus, leading to biallelically, paternally, and/or maternally expressed transcripts from both the sense and antisense strands. Expression from this locus is tightly regulated by epigenetic mechanisms and cis-acting regulatory elements, including non-coding RNA molecules. *GNAS* encodes Gsα, one of the subunits of the heterotrimeric stimulatory G protein, which is essential for the actions of numerous agonists. Several human disorders are caused by heterozygous mutations that affect Gsα expression and/or activity, and consistent with the tissue-specific paternal silencing of Gsα expression, these disorders are inherited in a parent-of-origin specific manner. In contrast to Gsα, the roles of most of the other *GNAS* products remain unknown at the cellular and molecular level, even though the biological significance of these products are clear from gene knockout studies. Depending on their parental origin, mutations that affect the Gsα transcript also disrupt some of the additional *GNAS* transcripts, such as XLαs. It is likely that the disruption of these additional gene products or alterations in the balance between the expression of Gsα and these proteins contributes to disease pathogenesis. It will therefore be important to investigate the factors regulating the expression of each *GNAS* product and to determine their cellular actions. Consequently, we will know more about the biological role of this gene locus and improve our understanding of the molecular mechanisms underlying the *GNAS*-related disorders. In addition, we will likely gain further insights into the functional significance of complex genes in the genome.
